# Compartmental surgery for T4b oral squamous cell carcinoma involving the masticatory space

**DOI:** 10.1097/MOO.0000000000000958

**Published:** 2024-01-08

**Authors:** Davide Mattavelli, Claudia Montenegro, Cesare Piazza

**Affiliations:** Department of Otorhinolaryngology – Head and Neck Surgery, ASST Spedali Civili of Brescia, University of Brescia, School of Medicine, Brescia, Italy

**Keywords:** compartmental surgery, masticatory space, oral cancer, T4b, transnasal endoscopic approach

## Abstract

**Purpose of review:**

This review aims to describe the oncological outcomes of T4b oral squamous cell carcinomas (OSCC) with masticatory space involvement as well as the surgical approaches that are able to achieve compartmental ‘en bloc’ resection of these lesions.

**Recent findings:**

The masticatory space is subdivided into infra-notch and supra-notch spaces according to the axial plane passing through the mandibular notch between the coronoid process and the condyle neck. Compartmental resection for T4b OSCC with masticatory space invasion can be successfully achieved via purely external approaches or combining external and transnasal endoscopic routes. Infra-notch T4b OSCC showed survival outcomes comparable to T4a OSCC, thus prompting treatment with curative intent.

**Summary:**

Compartmental resection of the masticatory space is technically feasible with comprehensive control of tumour margins. Use of a transnasal endoscopic anterior route within a multiportal approach may provide better control of margins at the level of the pterygo-maxillary fissure. Equivalent survival outcomes between T4a and infra-notch T4b OSCC are reported. Thus, a downstaging of the latter to T4a is advisable and compartmental surgery of such advanced lesions could be considered as a first-line treatment option in selected patients.

## INTRODUCTION

Oral squamous cell carcinoma (OSCC) is the 13th most common cancer worldwide [1]. The tongue and floor of mouth are the most common subsites in Europe and Northern America [2], while in Asia, where chewing tobacco, betel, and areca nut are still extremely popular, the buccal mucosa is the predominant subsite involved [3]. A critical, although rare, route of spread for OSCC is represented by the infratemporal fossa and masticatory space. Tumours originating from the upper/lower retromolar trigones and posterior buccal mucosa are at a higher risk of diffusion towards such an anatomical area in view of its close proximity. According to the American Joint Committee on Cancer (AJCC) [4], OSCC with involvement of the MS is staged as T4b (unresectable disease), together with those encasing the internal carotid artery or infiltrating the skull base and/or pterygoid plates. However, in the last two decades, increasing evidence has shown that selected T4b OSCC with involvement of the masticatory space can be treated with upfront surgery and successfully resected, with oncological outcomes that are comparable to those commonly observed for T4a cancers.

The idea of compartmental resection, borrowed from muscolo-skeletal sarcoma surgery, has been already extensively applied to OSCC, particularly for tongue and floor of the mouth tumours [5–11]. Different from conventional wide local excision, compartmental resection encompasses the removal of an entire anatomic unit in which the OSCC is contained along with all the related structures such as nerves, vessels, and muscles potentially representing routes for tumour spread. Thus, surgical resection by every compartmental approach is mostly anatomy-driven rather than cancer-driven, which leads to a more standardized, consistent and reproducible procedure in everyday clinical practice, with more desirable oncological outcomes [12].

The present review is aimed at describing the surgical approaches used for masticatory space compartmental resection, reporting the oncological outcomes obtained in the literature within this subset of T4b OSCC. 

**Box 1:**
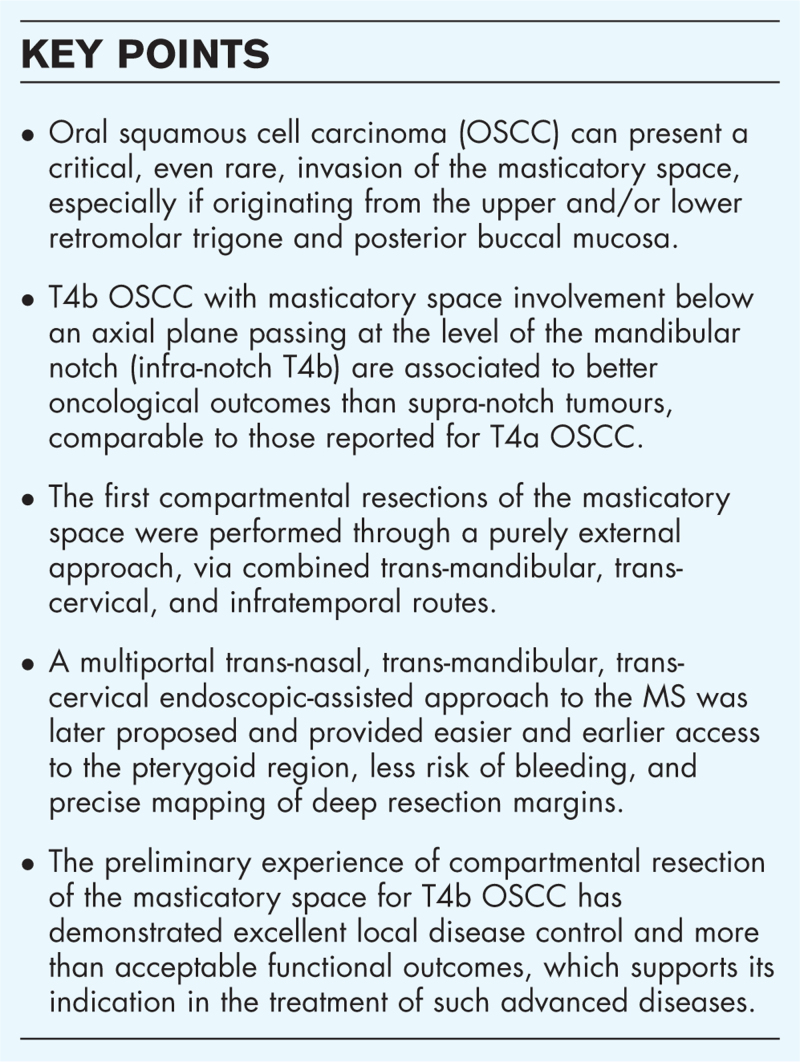
no caption available

## ANATOMY OF THE MASTICATORY SPACE

The masticatory space is an anatomically complex and deeply located region of the maxillo-facial skeleton. It is defined as a large, antero-lateral space of the supra-hyoid neck, extending from the mandibular angle to the high parietal calvarium, the greater wing of the sphenoid and temporal bones [13]. It contains the masticatory muscles (i.e., the masseter, medial, lateral pterygoid, and temporal muscles), ramus of the mandible, pterygoid plates, mandibular nerve, and internal maxillary artery with their branches. The masticatory space is contained in a single fascial system (the superficial layer of the deep cervical fascia, also called the investing fascia), and therefore represents a single anatomic compartment. In fact, the investing fascia splits along the inferior mandible in two components: the medial one runs along the deep surface of the pterygoid muscles and inserts on the inferior aspect of the skull base medial to the foramen ovale, while the lateral slip covers the surface of the masseter muscle, attaching to the zygomatic arch, and continues cephalad into the temporalis fascia to the top of the supra-zygomatic masticatory space [13–16]. The two fascial layers fuse at the anterior and posterior borders of the mandibular ramus, defining a closed, single anatomical unit.

## PROGNOSTIC SUBCLASSIFICATION OF T4b ORAL CANCER INVOLVING THE MASTICATORY SPACE

Liao *et al*. [17] proposed to subdivide the masticatory space according to an axial plane passing at the level of the mandibular notch: OSCC extending above this plane were defined as supra-mandibular notch tumours (supra-notch T4b, or SN–T4b), while the others were termed infra-notch T4b (IN–T4b). The latter were associated with a better oncological outcome with 5-year overall survival (OS) and disease-free survivals (DFS) of 55.3 and 64.7% for IN–T4b compared to 14.3 and 21.4% for SN–T4b, respectively [17]. In accordance with this evidence, Pillai *et al.*[18] found that tumours involving the lateral pterygoid muscle (and therefore anatomically defined as SN–T4b) had a poorer outcome (2-year DFS of 66 and 43% without and with lateral pterygoid muscle involvement, respectively). Moreover, a recent meta-analysis compared the oncological outcomes of surgically treated IN–T4b and T4a OSCC [19^▪▪^] tumours interestingly found no significant difference in OS, disease-specific survival (DSS), DFS, or local control among the two groups, which supports the downstaging of IN–T4b to T4a. In particular, for IN–T4b, the 2 and 5-year OS were 59.3 and 53.2%, 2 and 5-year DFS 57.9 and 48.4%, and 2 and 5-year local control 47 and 56% [19^▪▪^]. Similar data were reported in the study of Kang *et al.*[20] who compared the clinical outcomes of a cohort of Taiwanese patients affected by pT4a and pT4b OSCC undergoing surgical treatment. The authors reported no significantly different oncologic outcomes between pT4b and pT4a OSCC after propensity score matched analysis.

Trivedi *et al.*[21–23] highlighted this concept by proposing a classification of masticatory space invasion into three classes: low masticatory space (involvement of medial pterygoid, masseter muscles, and ascending ramus of the mandible), intermediate masticatory space (lateral pterygoid, temporalis muscle, and lower half of pterygoid plates), and high masticatory space (involvement of pterygo-maxillary fissure and intra-cranial extension). Class I corresponds to the infra-notch masticatory space, while class II and III to low and high supra-notch masticatory space, respectively.

## SURGICAL RESECTION OF TUMOURS OF THE MASTICATORY SPACE

Upfront surgery is the mainstay of treatment for OSCC. The rational to pursue a radical resection even in T4b OSCC involving the masticatory space is three-fold: regional and distant spread is less frequent than in other OSCC subsites (i.e., those involving the tongue and floor of mouth) [24,25]; local control is paramount to achieve cure or, at least, prolonged palliation with acceptable quality of life; and as previously shown, in selected T4b (i.e., IN–T4b) survival is comparable to T4a, which prompts for aggressive loco-regional treatments. In accordance, increasingly promising data on outcomes after surgery for T4b cancers have been reported after the first study described by Liao *et al.*[26].

From a surgical perspective, to ensure a well tolerated approach to the masticatory space, the surgeon must have a thorough understanding of its detailed anatomy. Furthermore, resection of the masticatory space is challenging in view of limited surgical corridor, abundant bleeding (mostly from the pterygoid venous plexus), and consequent difficulty in obtaining an adequate three-dimensional clearance of surgical margins. The reported rate of positive margins in T4b OSCC is, however, overall comparable to those observed for T4a, further reinforcing the feasibility of the resection of these ‘inoperable’ tumours. The close/positive margin rates in the series from Liao *et al.*[27] for T4a and T4b were, in fact, 13 and 14.6%, respectively, while in the study by Mair *et al.*[28] were 7.4 and 12%, respectively. Using compartmental resection through a purely external approach, Trivedi *et al*. [23] reported a 6.7% rate of positive margins, highlighting the fact that the pterygo-maxillary fissure is a region at higher risk of positive margins. In the study by Pillai *et al.*[18], margin positivity rates were 0.9% for T4a and 6.8% for T4b lesions. The preliminary clinical experience by Schreiber *et al.*[29] using an endoscopic-assisted compartmental resection reported negative margins in all three patients treated. In contrast to these results, the study by Baddour *et al.*[30] reported a high rate of close margins of 96% in patients with T4b tumours. This could be explained by the specimen-driven margin approach used by Baddour *et al.*[30], instead of a tumour-bed-driven technique; indeed, the authors reported that the compartmental resection as proposed by Trivedi *et al.*[22] was apparently related to improved clearance of margins.

Finally, Thiagarajan *et al.*[31] compared the rates of close/positive margins between patients treated by upfront surgery and those treated by neo-adjuvant chemotherapy (NACT) followed by surgery, and found that the rate was substantially higher in the former (49 vs. 8%). The use of NACT for advanced OSCC still remains controversial however [32–34]. The response rate to chemotherapy reported in the literature, in fact, varies within a wide range, between 28% [33] and 82% [34]. A retrospective pair-matched analysis comparing treatment outcomes and recurrence patterns in patients with cT4a/T4b OSCC undergoing upfront surgery or NACT indicated an OS benefit for the latter group of patients with cT4b disease; in contrast, a benefit of upfront surgery was found for cT4a lesions [31]. For T4b OSCC, the superiority of surgery compared to nonsurgical treatment was demonstrated with a better OS rate for surgical treated patients [35^▪▪^]. This finding may pave the way for a new treatment paradigm for cT4b OSCC, although further validation in a prospective, randomized trial is warranted.

## COMPARTMENTAL RESECTION OF THE MASTICATORY SPACE

The first description of the resection of an OSCC extending to the masticatory space dates to 1959 when Barbosa *et al.*[36] reported a surgical approach for retromolar trigone carcinoma including mandibulectomy with en bloc removal of the pterygoid and masseter muscles along with ipsilateral neck dissection. This approach was later popularised by Kowalsky *et al.*[37] who referred to it as the ‘commando’ operation.

The first truly compartmental resection of the masticatory space was then described by Trivedi *et al.* in 2012 [22,23]. It was performed through a purely external approach, via combined trans-mandibular, trans-cervical, and infratemporal routes [22,23], and included segmental mandibulectomy, resection of the upper alveolus, skin (if involved), masseter, medial and lateral pterygoid muscles with pterygoid plates and part of the temporalis muscle, and sacrificing the mandibular nerve (from the foramen ovale to the lingual and inferior alveolar nerves). In this way, the authors emphasized the ease of dissection offered by an approach in which it was carried out in a wider plane around the muscles rather through them. With such a compartmental resection, three out of 45 patients (6.6%) [23] had positive margins at the anterior mandibulectomy margin (in one case) and along the pterygo-maxillary fissure (in two cases) [22]. In this way, the major limitation of purely external approaches, that is, the limited access to the pterygoid process and pterygo-maxillary fissure, which are considered two of the most critical targets in which to obtain negative margins, became evident. To overcome this limitation, a multiportal (trans-nasal, transoral, and trans-cervical) endoscopic-assisted approach to the masticatory space was later proposed [29].

## ENDOSCOPIC-ASSISTED MULTI-PORTAL COMPARTMENTAL RESECTION OF THE MASTICATORY SPACE

**FIGURE 1:**
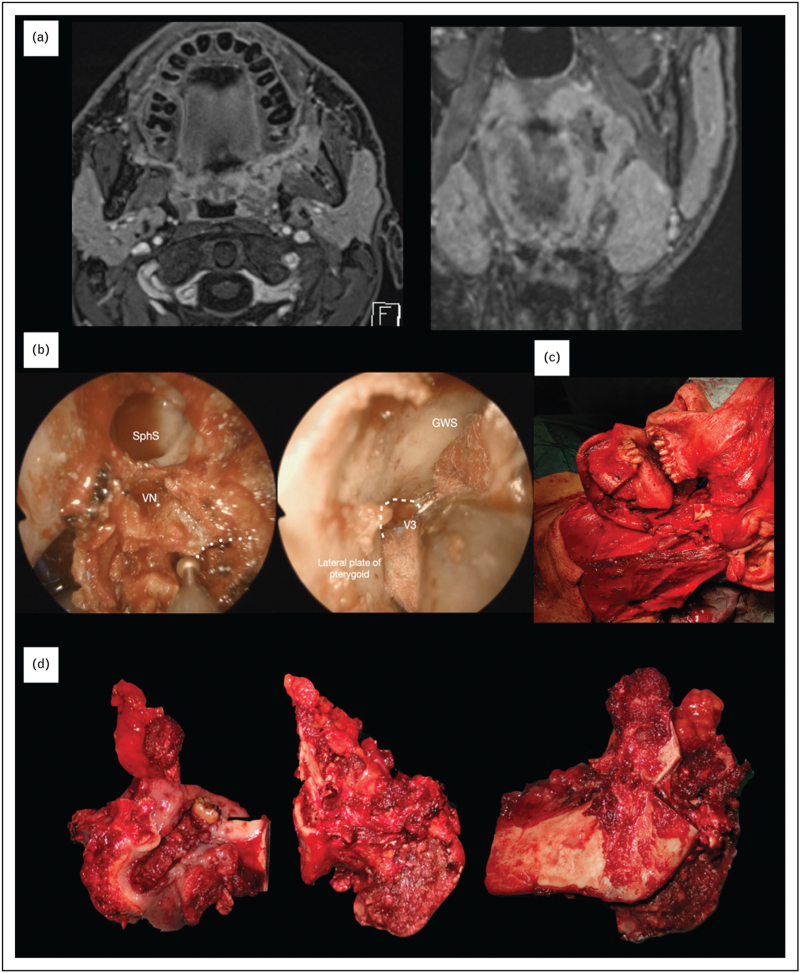
A 42-year-old male smoker referred to us complaining a growing hard mass of the left inferior retromolar trigone. (a) MR showed involvement of the masticatory space (MS) and regional lymph nodes with radiologic evidence of extranodal extension. PET scan was negative for distant metastases (cT4b N3b M0). An endoscopic-assisted multiportal trans-nasal (b), trans-mandibular and trans-cervical (c) compartmental resection of the MS with left modified radical neck dissection type III, right selective neck dissection (level I-III) and reconstruction with antero-lateral thigh free flap was performed. En bloc resection of the specimen (d) allowed accurate evaluation of the surgical margins both intraoperatively and during histopathologic examination. The final histopathologic report staged the tumour as pT4b N3b Lv1 Pn1 with negative margins. Adjuvant chemo-radiotherapy was administered. After 4 months distant metastases (bone and lung) were detected, and the patient was treated with immunotherapy. He is alive with stable disease 24 months after surgery. Dashed line, foramen ovale; dotted line, greater wing of the sphenoid bone; GWS, greater wing of the sphenoid bone; SphS, sphenoid sinus; V3, mandibular nerve; VN, vidian nerve.

The endoscopic-assisted multiportal compartmental resection described by Schreiber *et al.*[29] consisted of three phases: an endoscopic endonasal initial phase followed by a trans-mandibular, trans-cervical approach with a multiportal en bloc final tumour resection. The final resection is similar to that proposed by Trivedi and coworkers, while the endoscopic endonasal initial approach essentially presents the following advantages: easier and earlier access to the pterygoid region, pterygo-maxillary fissure, and middle skull base via the trans-nasal route, allowing optimal visualisation of critical anatomical structures (blood vessels) and adequate check of the most difficult pathways of tumour spread (such as endocranial diffusion via the branches of the trigeminal nerve); complete oversight of the surgical field, eliminating blind spots and promptly addressing the pterygo-maxillary fissure; endoscopic magnification, with precise mapping of deep resection margins via frozen sections; and in case of tumours with border-line resectability, early access to these critical regions may favour intraoperative confirmation of the palliative scenario and allow immediate discontinuation of the procedure without any major associated sequelae (’primum non nocere’). Figure 1 shows the radiological and surgical details of one patient treated with such a multiportal en bloc compartmental resection of a T4b OSCC at our Department.

## POSTOPERATIVE COMPLICATIONS

Different postoperative complications have been reported in the literature, depending on the type and extent of surgery performed, especially for external approaches. The mean surgical rate of complications reported is 37.9% (22 of 58 patients) [22,29,30].

Baddour *et al.*[30] reported both medical and surgical complications (72 and 48%, respectively): the two most common medical complications were postoperative delirium (28%) and severe anaemia requiring transfusion (48%), while the most common surgical complication was oro-cutaneous fistula (12%). Due to the limited intraoperative visualisation and high risk of copious bleeding caused by the pterygoid venous plexus, especially in patients treated with a nonendoscopic-assisted approach, other postoperative surgical complications were secondary haemorrhage from masticatory space area (controlled with ligation of the external carotid artery) and venous thrombosis of free flap used for reconstruction [22].

Bleeding is not a complication among patients treated by endoscopic-assisted multiportal compartmental resection [29], but one experienced a postoperative oro-cervical fistula with neck abscess, while one free flap used for a failed reconstruction and needed a second free flap. The reconstructive phase after such a multiportal resection must be carefully addressed and encompasses special challenges in order to reduce to a minimum the occurrence of oro-cutaneous fistulas with ensuing deep cervical spaces abscess formation. In fact, the harvested flap must be large enough to separate the nasopharynx, oropharynx and oral cavity from the neck and above mentioned deep maxillo-facial regions. Suture of the skin with the nasopharyngeal mucosa is somewhat difficult and can leave minor gaps just below the skull base and the masticatory space. For this reason, an adequate amount of muscle or fat tissue must fill the surgical bed in a three-dimensional fashion, and antero-lateral thigh, latissimus dorsi, or rectus abdominis are the most suitable donor sites to achieve these goals. The vascular pedicle does not need to be excessively long since the distance between the recipient surgical bed and the external carotid system/internal or external jugular system is usually within 6--7 cm. The issue of mandibular reconstruction, on the other hand, must be balanced according to these overall requirements. A fibular flap usually does not contain per se enough soft tissues to reconstruct the mucosal defect and fill the dead space created by masticatory space clearance. In young, dentate patients requiring full oral rehabilitation, a double free flap should be therefore planned. In this case, we usually associate osteo-muscular fibular and antero-lateral thigh free flaps. In elderly or edentulous patients, in contrast, no mandibular reconstruction or a simple reconstruction plate surrounded by soft tissues free flaps alone may equally allow an adequate reconstruction.

## FUNCTIONAL OUTCOMES

This kind of surgery nearly always requires temporary tracheotomy and nasogastric or percutaneous gastrostomy feeding tube for at least 7–10 days postoperatively. According to data reported in the literature, Baddour *et al.*[30] performed tracheotomy in 92% of their patients, while 39.1% remained tracheostomy-dependent at more than 30 days postoperatively. Likewise, the majority of patients required gastrostomy tubes (84%), with 89% of those patients continuing to require enteral access at more than 6 months after surgery. Trivedi *et al.*[22] stated that all 30 patients in their series could at least eat soft diet, and most attained acceptable communication skills, lip continence and cosmesis. After endoscopic-assisted multiportal compartmental resection [29], all patients resumed oral feeding and closed tracheostomy before discharge. Only one patient, during follow-up, needed a percutaneous gastrostomy to integrate oral intake due to mild dysphagia after adjuvant chemoradiation therapy.

## CONCLUSION

pT4b OSCC for invasion of the masticatory space can be surgically treated with reasonable oncological outcomes. In case of an IN–T4b tumour, in fact, survivals have been demonstrated to be at least comparable to those observed in T4a OSCC. However, to reach these results, the concept of masticatory space compartmental surgery, similar to what described for T3-T4 tongue OSCC, must be implemented. Several preliminary experiences with external or multiportal approaches have demonstrated its feasibility from a technical standpoint and conveyed promising oncological and functional outcomes. However, these resections are complex and burdened by potentially serious perioperative and postoperative complications. Their centralisation in high volume referral centres with adequate endoscopic, open and reconstructive expertise is mandatory. Even if more prospective studies are required, the scientific community should contemplate subclassifying T4b OSCC, trying to better define which group of patients a curative approach is most indicated. According to the published evidence, a reclassification of current IN–T4b patients to T4a OSCC should be considered and treatments with curative intent pursued in selected patients.

## Acknowledgements

*None.*

### Financial support and sponsorship

*None.*

### Conflicts of interest

*There are no conflicts of interest.*

## REFERENCES AND RECOMMENDED READING

Papers of particular interest, published within the annual period of review, have been highlighted as:

▪ of special interest
▪▪ of outstanding interest

## References

[1] Oral health. https://www.who.int/news-room/fact-sheets/detail/oral-health. [Accessed 24 October 2023]

[2] Warnakulasuriya S. Global epidemiology of oral and oropharyngeal cancer. Oral Oncol 2009; 45:309–316. 18804401 10.1016/j.oraloncology.2008.06.002

[3] India State-Level Disease Burden Initiative Cancer Collaborators. The burden of cancers and their variations across the states of India: the Global Burden of Disease Study 1990–2016. Lancet Oncol 2018; 19:1289–1306. 30219626 10.1016/S1470-2045(18)30447-9 PMC6167407

[4] Oral cavity and oropharyngeal cancer stages. https://www.cancer.org/cancer/types/oral-cavity-and-oropharyngeal-cancer/detection-diagnosis-staging/staging.html. [Accessed 24 October 2023]

[5] Calabrese L Giugliano G Bruschini R. Compartmental surgery in tongue tumours: description of a new surgical technique. Acta Otorhinolaryngol Ital 2009; 29:259–264. 20162027 PMC2821124

[6] Tagliabue M Gandini S Maffini F. The role of the T-N tract in advanced stage tongue cancer. Head Neck 2019; 41:2756–2767. 30942940 10.1002/hed.25761

[7] Missale F Marchi F Iandelli A. Oncological outcomes of compartmental surgery and wide local excision in oral tongue and floor of the mouth cancer. Oral Oncol 2022; 135:106210. 36306673 10.1016/j.oraloncology.2022.106210

[8] Calabrese L Tagliabue M Grammatica A. Compartmental tongue surgery for intermediate-advanced squamous cell carcinoma: a multicentric study. Head Neck 2023; 45:2862–2873. 37727894 10.1002/hed.27517

[9] Grammatica A Piazza C Montalto N. Compartmental surgery for oral tongue cancer: objective and subjective functional evaluation. Laryngoscope 2021; 131:E176–E183. 32239760 10.1002/lary.28627

[10] Piazza C Grammatica A Montalto N. Compartmental surgery for oral tongue and floor of the mouth cancer: oncologic outcomes. Head Neck 2019; 41:110–115. 30536781 10.1002/hed.25480

[11] Calabrese L Bruschini R Giugliano G. Compartmental tongue surgery: long term oncologic results in the treatment of tongue cancer. Oral Oncol 2011; 47:174–179. 21257337 10.1016/j.oraloncology.2010.12.006

[12] Grammatica A Piazza C Ferrari M. Step-by-step cadaver dissection and surgical technique for compartmental tongue and floor of mouth resection. Front Oncol 2021; 11:613945. 33968719 10.3389/fonc.2021.613945 PMC8104033

[13] Galli F Flor N Villa C. The masticator space. Value of computed tomography and magnetic resonance imaging in localisation and characterisation of lesions. Acta Otorhinolaryngol Ital 2010; 30:94–99. 20559479 PMC2882142

[14] Harnsberger HR. Handbook of head and neck imaging. 1995; USA: Mosby, 580 p.

[15] Koch BL Hamilton BE Hudgins PA Harnsberger HR. Diagnostic imaging: head and neck. 2016; Amsterdam, Netherlands: Elsevier, 1352 p.

[16] Fernandes T Lobo JC Castro R. Anatomy and pathology of the masticator space. Insights Imaging 2013; 4:605–616. 23888350 10.1007/s13244-013-0266-4 PMC3781239

[17] Liao CT Ng SH Chang JTC. T4b oral cavity cancer below the mandibular notch is resectable with a favorable outcome. Oral Oncol 2007; 43:570–579. 16996777 10.1016/j.oraloncology.2006.06.008

[18] Pillai V Yadav V Kekatpure V. Prognostic determinants of locally advanced buccal mucosa cancer: do we need to relook the current staging criteria? Oral Oncol 2019; 95:43–51. 31345393 10.1016/j.oraloncology.2019.05.021

[19▪▪] Rao KN Arora R Dange P. A meta-analysis of surgical outcomes of T4a and infranotch T4b oral cancers. Oncol Ther 2023; 11:461–480. 37804420 10.1007/s40487-023-00246-3 PMC10673764

[20] Kang CJ Wen YW Lee SR. Clinical outcomes of patients with pT4a and pT4b oral cavity squamous cell carcinoma who had undergone surgery: results from a Taiwanese registry-based, nationwide cohort study. Oral Oncol 2022; 126:105750. 35123256 10.1016/j.oraloncology.2022.105750

[21] Trivedi N. Atlas for head and neck cancer surgery: the Compartment surgery for resection in 3-D. 2015; Springer New Delhi: Springer, 2015.

[22] Trivedi NP Kekatpure V Kuriakose MA. Radical (compartment) resection for advanced buccal cancer involving masticator space (T4b): our experience in thirty patients. Clin Otolaryngol dicembre 2012; 37:477–483. 10.1111/j.1749-4486.2012.02529.x 23253342

[23] Trivedi NP Kekatpure VD Shetkar G. Pathology of advanced buccal mucosa cancer involving masticator space (T4b). Indian J Cancer 2015; 52:611–615. 26960493 10.4103/0019-509X.178410

[24] Pathak KA Gupta S Talole S. Advanced squamous cell carcinoma of lower gingivobuccal complex: patterns of spread and failure. Head Neck 2005; 27:597–602. 15825204 10.1002/hed.20195

[25] Trivedi NP. Oral cancer involving masticator space (T4b): review of literature and future directions. Head Neck 2018; 40:2288–2294. 29756367 10.1002/hed.25211

[26] Liao CT Chang JTC Wang HM. Surgical outcome of T4a and resected T4b oral cavity cancer. Cancer 2006; 107:337–344. 16770782 10.1002/cncr.21984

[27] Liao CT Lee LY Hsueh C. Comparative outcomes in oral cavity cancer with resected pT4a and pT4b. Oral Oncol 2013; 49:230–236. 23063612 10.1016/j.oraloncology.2012.09.010

[28] Mair MD Sawarkar N Nikam S. Impact of radical treatments on survival in locally advanced T4a and T4b buccal mucosa cancers: selected surgically treated T4b cancers have similar control rates as T4a. Oral Oncol 2018; 82:17–22. 29909893 10.1016/j.oraloncology.2018.04.019

[29] Schreiber A Mattavelli D Accorona R. Endoscopic-assisted multiportal compartmental resection of the masticatory space in oral cancer: anatomical study and preliminary clinical experience. Oral Oncol 2021; 117:105269. 33827034 10.1016/j.oraloncology.2021.105269

[30] Baddour HM Ochsner MC Patel MR. Surgical resection is justifiable for oral T4b squamous cell cancers with masticator space invasion. Laryngoscope 2021; 131:E466–E472. 32460370 10.1002/lary.28725 PMC7704866

[31] Thiagarajan S Dhar H Bhattacharjee A. Patterns of failure and outcomes in cT4 Oral squamous cell carcinoma (OSCC) undergoing upfront surgery in comparison to Neo-Adjuvant Chemotherapy (NACT) followed by surgery: a matched pair analysis. Oral Oncol 2020; 100:104455. 31739192 10.1016/j.oraloncology.2019.104455

[32] Okura M Hiranuma T Adachi T. Induction chemotherapy is associated with an increase in the incidence of locoregional recurrence in patients with carcinoma of the oral cavity: results from a single institution. Cancer 1998; 82:804–815. 9486567

[33] Joshi A Patil VM Noronha V. Is there a role of induction chemotherapy followed by resection in T4b oral cavity cancers? Indian J Cancer 2013; 50:349–355. 24369216 10.4103/0019-509X.123627

[34] Licitra L Grandi C Guzzo M. Primary chemotherapy in resectable oral cavity squamous cell cancer: a randomized controlled trial. J Clin Oncol 2003; 21:327–333. 12525526 10.1200/JCO.2003.06.146

[35▪▪] Lin NC Hsu JT Chen MYC Tsai KY. Survival and clinicopathological characteristics of cT4b oral squamous cell carcinoma based on different treatment modalities: a single-center retrospective study. Medicine (Baltimore) 2022; 101:e29285. 35583539 10.1097/MD.0000000000029285 PMC9276147

[36] Barbosa JF. Cancer of the retromolar area; a study of twenty-eight cases with the presentation of a new surgical technique for their treatment. AMA Arch Otolaryngol 1959; 69:19–30. 13605356 10.1001/archotol.1959.00730030023004

[37] Kowalski LP Hashimoto I Magrin J. End results of 114 extended «commando» operations for retromolar trigone carcinoma. Am J Surg 1993; 166:374–379. 8214296 10.1016/s0002-9610(05)80336-8

